# How Are Non-Medical Settlement Service Organizations Supporting Access to Healthcare and Mental Health Services for Immigrants: A Scoping Review

**DOI:** 10.3390/ijerph19063616

**Published:** 2022-03-18

**Authors:** Ayesha Ratnayake, Shahab Sayfi, Luisa Veronis, Sara Torres, Sihyun Baek, Kevin Pottie

**Affiliations:** 1 Faculty of Health Sciences, University of Ottawa, Ottawa, ON K1N 6N5, Canada; 2 Faculty of Science, University of Ottawa, Ottawa, ON K1N 6N5, Canada; ssayf086@uottawa.ca; 3 Department of Geography, Environment and Geomatics, University of Ottawa, Ottawa, ON K1N 6N5, Canada; lveronis@uottawa.ca; 4 School of Social Work, Laurentian University, Sudbury, ON P3E 2C6, Canada; storres@laurentian.ca; 5 Faculty of Medicine, University of Ottawa, Ottawa, ON K1N 6N5, Canada; sbaek040@uottawa.ca; 6 C.T. Lamont Primary Health Care Research Centre, University of Ottawa, Ottawa, ON K1N 6N5, Canada; kpottie@uwo.ca; 7 Department of Family Medicine, Western University, London, ON N6A 3K7, Canada

**Keywords:** immigrants, refugees, primary healthcare access, settlement service organizations, health equity

## Abstract

Following resettlement in high-income countries, many immigrants and refugees experience barriers to accessing primary healthcare. Local non-medical settlement organizations, such as the Local Immigration Partnerships in Canada, that support immigrant integration, may also support access to mental health and healthcare services for immigrant populations. This scoping review aims to identify and map the types and characteristics of approaches and interventions that immigrant settlement organizations undertake to support access to primary healthcare for clients. We systematically searched MEDLINE, Social Services Abstracts, CINAHL, and PsycInfo databases from 1 May 2013 to 31 May 2021 and mapped research findings using the Social-Ecological Model. The search identified 3299 citations; 10 studies met all inclusion criteria. Results suggest these organizations support access to primary healthcare services, often at the individual, relationship and community level, by collaborating with health sector partners in the community, connecting clients to health services and service providers, advocating for immigrant health, providing educational programming, and initiating community development/mobilization and advocacy activities. Further research is needed to better understand the impact of local non-medical immigrant settlement organizations involved in health care planning and service delivery on reducing barriers to access in order for primary care services to reach marginalized, high-need immigrant populations.

## 1. Introduction

Given the growing numbers of culturally and linguistically diverse newcomers settling in Canada annually, pressure is being placed on provincial and federal governments to involve local non-medical immigrant settlement organizations in the development of accessible equitable healthcare and welfare services to meet the complex needs of expanding marginalized populations such as immigrants and refugees [1,2]. Asylum seekers also often have significant healthcare needs, due to premigration and post-migration experiences, yet tend to have low participation in primary healthcare systems [3,4]. Further, there is a growing need to support migrants’ access to mental health services, as research has shown that they are at a higher risk for mental health problems compared to the general population but are less likely to seek care [5].

The focus on access to quality primary healthcare services is important, since these populations may be vulnerable and often experience considerable barriers to accessing quality primary healthcare, including limited English language proficiency, culturally inappropriate care and varying health beliefs, transportation difficulties, a general lack of social support, health system and health literacy issues, and high service costs [2,6,7,8,9]. Additionally, many health professionals report increased complexity on their end when serving migrant and refugee clients, relating to factors such as language interpretation difficulties, social determinants of health that require a multi-sector response, as well as difficulties for clients in understanding various health service entitlements [10]. Since the COVID-19 pandemic reduced community settlement services, primary care practitioners reported a corresponding reduction in access to primary healthcare for refugees and newcomers [11].

Local non-medical immigrant settlement organizations that support immigrant integration can facilitate collaborative efforts to increase access to mental health and healthcare services for migrants. They can also support information sharing by acting as a platform to connect various actors horizontally across sectors and vertically within sectors. These partnerships create a social space where civil society, businesses, private-sector stakeholders, local municipalities, and other stakeholders can discuss priority issues [12]. Variations of local immigrant settlement organizations and partnerships can be found globally; for example, the Strategic Migration Partnership in London, Local Immigration Partnerships (LIPs) in Canada, and the Mayor’s Offices for Immigrant Affairs in Chicago, are a few well-established groups [12]. In Canada, LIPs play an essential role in immigrant settlement and integration [13,14]. Led by municipal or regional governments, or community organizations, LIPs are broad, cross-sectoral convening bodies that integrate newcomer needs into a city’s community planning [13]. The LIPs play a central role in supporting immigrant populations by increasing local stakeholders’ engagement in newcomers’ integration processes, supporting community-level research and planning, and improving service coordination [15].

There has been some mixed-methods research, conducted with providers, refugees and interpreters, to gain insight into how these non-medical immigrant settlement organizations collaborate with the health sector [16]; however, to the best of our knowledge, the ways and opportunities through which these non-medical immigrant settlement organizations are supporting immigrants’ access to mental health and other healthcare services have not been thoroughly examined or defined [17]. We aimed to address this knowledge gap by establishing how these “untapped resource” organizations contribute to improving immigrant access to primary health care services to create more health-enhancing environments for communities and marginalized populations. To guide our review, we asked the following research question: How do non-medical, local immigrant settlement organizations support access to healthcare services (i.e., primary healthcare services and/or specialized healthcare services) for immigrant populations in high-income countries? Our objectives were to identify and to map the types of approaches and interventions that non-medical immigrant settlement organizations use to support primary care access for immigrants. To inform our analysis and mapping approach, we adopted the social-ecological model [18].

## 2. Materials and Methods

### 2.1. Protocol

We developed a protocol for this scoping review using Arksey and O’Malley’s 2005 five-stage methodological framework [19], and refined stage 5 as per recommendations made by the Joana Briggs Institute [20]. This scoping review included the following five key stages: (1) identifying the research question; (2) identifying relevant studies; (3) study selection; (4) charting the data; and (5) collating, summarizing, and reporting the results. To map and organize our data, we used an Excel data extraction sheet informed by the social-ecological model [18]. To report our findings, we replaced Arksey and O’Malley’s approach with the Preferred Reporting Items for Systematic Reviews and Meta-Analyses Scoping Review (PRISMA-ScR) checklist (see Additional File S1 in Supplementary Materials) [21]. The final version of the protocol is available upon request.

### 2.2. Data Sources and Search Strategy

In consultation with an expert health sciences librarian (LS), we developed a strategy to systematically search—using keywords, MeSH terms, major subject headings and/or the thesaurus functions—the following four electronic databases from 1 May 2013 to 31 May 2021: MEDLINE, Social Services Abstracts, PsycInfo, and Cumulative Index to Nursing and Allied Health Literature (CINAHL). An expert social sciences research librarian (PL) reviewed our social services abstracts search strategy, which consisted of terms such as refugee, immigrant, asylum seeker, local, community, partnership, organization, collaboration, primary health care, clinical care, health services accessibility, mental health services, Canada, United States, and Australia; the search terms were combined using Boolean operators (see Additional File S2 in Supplementary Materials for complete search strategy). Moreover, the search query was tailored to the specific requirements of each database. Lastly, we scanned references of the included articles for any relevant studies.

### 2.3. Eligibility Criteria

We included articles that met the following criteria: (1) included refugee, asylum seeker, or immigrant populations; (2) described local non-medical immigrant settlement-type organizations that support immigrant access to primary or clinical health care services; and (3) were conducted in industrialized countries with demographic, economic, political, and social characteristics comparable to those of Canada, and that are ranked on healthcare system performance by the Commonwealth Fund (see Table 1 for full inclusion criteria) [22]. Moreover, we used the United Nations High Commissioner for Refugees (UNHCR) definitions for asylum seekers and refugees as criteria for paper inclusion, while relying on Statistics Canada’s definition for the term immigrant [23,24,25]. Specifically, we included studies that focused on refugees, asylum seekers, and immigrants 16 years of age and older; those that examined populations of any other age were excluded due to methodological challenges around the design, conduct and reporting of pediatric systematic reviews. For feasibility reasons, studies on undocumented migrants, transient migrant workers, foreign temporary workers, and foreign students were excluded. Organizations that did not conduct settlement-type work for immigrant populations, were not local, or were medical organizations were excluded. Lastly, countries that were not ranked by the Commonwealth Fund on healthcare system performance were excluded [22].

**Figure 1:**
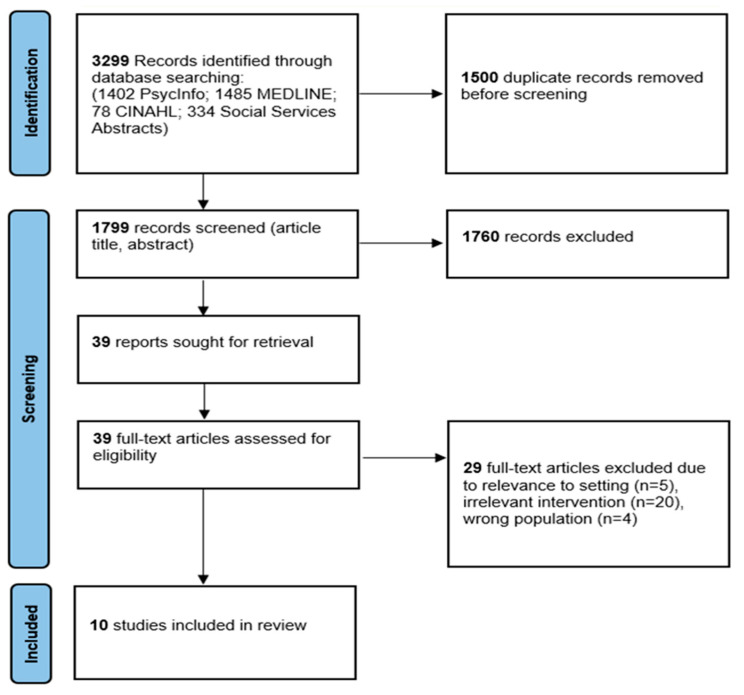
The Preferred Reporting Items for Systematic Reviews and Meta-Analyses (PRISMA) study flow diagram.

Due to resource constraints, we applied restrictions to select articles that were most relevant. Literature reviews were excluded since, by nature, they are not primary data research publications; gray literature was excluded because the diverse formats and audiences of these texts can present a significant challenge in a systematic search for peer-reviewed evidence. We also excluded studies that were published prior to the last 8 years after reviewing Waleed M. Sweileh et al.’s 2018 paper “Bibliometric analysis of global migration health research in peer-reviewed literature (2000–2016)” in BMC Public Health, since it analyzed peer-reviewed literature in global migration health published worldwide [27]. Based on two key findings from the Bibliometrics, we applied the assumption that much of the global migration health research performed from 2014 onwards has taken into consideration prior research in earlier years; these key findings are as follows: the Bibliometrics’ Figure 1 analysis demonstrates an up-tick in global migration health publications from 2014–2016 (approximately one third of the retrieved documents in the analysis were published in the last 3 years of the study); and the Bibliometric reference list includes publications that focused on access to healthcare services and community organization support for migrants that were published between 2015–2017—for example, Taylor J.’s 2017 systematic review of social determinants of health on access to healthcare [28]. Therefore, since this “explosion” of migrant access to healthcare research occurs around 2014, we decided to limit our study’s search to publications from 2013 and onwards.

### 2.4. Study Selection Process

Search results were imported into COVIDENCE, an online systematic review software [29]. The inclusion criteria were used for screening titles and abstracts during level 1 screening and reviewing full-text articles during level 2 screening. Two reviewers (AR and SS) independently screened the title and abstract of each article for inclusion. Reviewers connected with one another throughout the screening process to resolve conflicts and discuss any uncertainties that arose during the selection process. All articles deemed relevant after title and abstract screening were included for full-text screening. Using the same process, the two reviewers (AR and SS) subsequently screened the full text of potentially relevant articles to determine eligibility. Disagreements were resolved through discussion between the two reviewers. Once agreement was reached, the full-text articles chosen for inclusion in the study were reviewed for data extraction.

### 2.5. Data Extraction

A standardized data extraction template, informed by framework analysis using the social-ecological model, was developed with input from the entire review team [18]. We chose the social-ecological model because it is a commonly used population health framework to conceptualize health broadly, taking into consideration that health is affected by dynamic interactions among various personal and environmental factors [18]. At minimum, results for our study were extracted as they applied to the framework analysis (individual level, relationship level, community level, societal level) and study criteria. For all of the articles included in the final analysis, data were extracted on the following variables: (1) author and year of publication, (2) source origin (i.e., country where the study took place), (3) aim/purpose of the study, (4) list of organizations that participated in the study, (5) study population/sample size/study participant description (i.e., participant characteristics), (6) methodology, (7) intervention type, (8) concepts or phenomena of interest, (9) outcomes measured, and (10) key findings/author conclusions/implications. In order to ensure the validity of the data extraction form, it was piloted by two reviewers (SS and SB), and accuracy of the content was reviewed by a third reviewer (AR). For all articles, two reviewers extracted data in duplicate and independently (SS and SB). Results were compared and disagreements were resolved by discussion or with help from a third reviewer (AR).

### 2.6. Methodological Quality Appraisal

We did not appraise the methodological quality or risk of bias of the included articles, which is consistent with guidance on scoping review conduct [20]. As a scoping review, the purpose of this study was to aggregate the findings and present a mapping of the research rather than to evaluate the quality of the individual studies [19]. Therefore, a critical appraisal of the methods for the strength of the evidence was not performed.

### 2.7. Data Mapping and Synthesis

As Carroll et al., 2013 recommended, we used a framework analysis method to structure our results [30]. Specifically, the theoretical social-ecological model was applied to map and group findings into themes and identify and explain outliers [18]. Results are presented in a table summarizing the characteristics of included studies with narrative descriptions. We discuss the application of findings to the broader context and discussion on non-medical immigrant settlement organizations supporting access to healthcare service and provide conclusions/implications for policy research and practice. We also identify and discuss strengths and limitations of the scoping review.

## 3. Results

### 3.1. Literature Search

A total of 3299 records were identified through database searching. After removal of duplicate citations, 1799 records were screened by title and abstract. Title abstract screening resulted in the exclusion of 1760 records, leaving 39 potentially relevant full-text articles that were sought for retrieval and assessed for eligibility using the inclusion criteria. Figure 1 presents the details of the search process. From these, 29 full-text articles were further excluded due to relevance to setting, irrelevant intervention or wrong population. The remaining 10 articles were included in this review: Chadwick and Collins, 2015 (study 1) [31]; Cheng et al., 2019 (study 2) [32]; Frost et al., 2018 (study 3) [33]; Isaacs et al., 2013 (study 4) [34]; Isaacs et al., 2013 (study 5) [35]; Koehn et al., 2019 (study 6) [36]; McMurray et al., 2014 (study 7) [37]; Salami et al., 2019 (study 8) [38]; Torres et al., 2013 (study 9) [39]; and Torres et al., 2014 (study 10) [7]. Characteristics of included studies are summarized in Table 2.

### 3.2. Study Characteristics

Of the 10 articles, eight were carried out in Canada (study 1, 4–10), one was in the USA (study 3), and one was in Australia (study 2). Three studies were published in 2019 (study 2, 6, 8), one in 2018 (study 3), one in 2015 (study 1), two in 2014 (study 7, 10), and three in 2013 (study 4, 5, 9). Study designs included qualitative interviews (study 1, 3–6, 8–10), qualitative surveys (study 4, 5), quantitative survey analysis (study 1), intervention development/piloting (study 2), before/after repeated survey design (study 7), focus groups (study 6, 8), and other research methods such as direct observation, document/database analysis (study 9, 10). Local non-medical immigrant settlement organizations in the 10 studies were described as settlement service organizations (study 1), local settlement support agencies (study 2), local refugee resettlement agency (study 3), community-based organizations (study 4, 5, 9, 10), immigrant-serving agencies (study 6, 8), and local receiving center (study 7).

In the 10 articles reviewed, the local non-medical immigrant settlement organizations’ priority populations served included recent immigrants in small or large urban centers (study 1); asylum seekers newly released from detention (study 2) (note: 93% of the clients were men, 54% of clients were aged between 22 and 34 years, countries of origin included Afghanistan (30.4%), Sri Lanka (25.3%), Iran (19.2%), Pakistan (10.7%), Other (6%), Stateless (3.7%), Vietnam (3.3%), and Iraq (1.4%)); Burmese-speaking refugee women (study 3), recent immigrant families in urban centers (study 4, 5); Punjabi and Korean-speaking immigrants (study 6); government-assisted refugees (study 7) (note: study population included males (50.9%) and females (49.1%) with a large percentage under the age of 18 (49.2%), primarily coming from Northwest Africa, the Middle East, and Southeast Asia); immigrants and refugees (study 8); and at-risk immigrant and refugee women and their families (study 9, 10).

### 3.3. Approaches to Support Access to Primary Healthcare Services for Immigrants

The findings from our study are presented below according to the various levels of the Social-Ecological Model [18]. The first level, individual, identifies personal and biological factors that directly or indirectly impact health outcomes, while the second level, relationships, consists of close social environment factors that may influence the health outcomes of an individual. The community level of the Social-Ecological Model refers to the various factors associated with the setting in which a person goes about their daily life. Lastly, the societal level looks at broad social, economic, and political factors that influence a person’s health status [18].

#### 3.3.1. Individual Level

Two studies fell under this theme. Study 3 evaluated a pilot health education intervention delivered to Burmese-speaking refugee women, clients at a resettlement agency in Houston, Texas. Developed in partnership with the University of Texas Health Science Center, the intervention provided learning events to develop new skills to navigate health services, held discussions on health topics and question and answer (Q&A) sessions with medical providers, and disseminated health education resources. The increased opportunities to practice English and develop vocabulary allowed participants to be more confident in executing skills such as calling a doctor’s office to make appointments or taking the bus. The study noted that lack of compatibility and agency buy-in were two main barriers to creating a feasible and sustainable intervention.

Next, study 8 focused on service providers’ perceptions of immigrant and refugee access to and use of mental health services. Findings showed that immigrant-serving agencies played a significant role in identifying clients experiencing a crisis or struggling with mental health conditions and connecting these individuals to mental health services. Further, these providers also evaluated the fit of an interpreter or cultural broker (brokers provide education and cultural translation support) with a client. In terms of challenges, the participants noted a desire for increased mental health training on identifying client needs and referring clients to specialized mental health services.

#### 3.3.2. Relationship Level

Relationship level was examined in two studies. In study 5, the focus was on community-based organizations’ trust in the cultural competency of other local service providers and its influence on meeting the complex healthcare needs of recent immigrant families. Cultural competency in this study refered to the ability and preparedness of a service organization to understand and respond to the health needs of immigrant families. Competence trust among service organizations was key for families to have access to healthcare services, whereas a lack of trust led to constrained workflow within the system, more avoidance behavior, and less interaction. The study found settlement service organizations to be exemplars of cultural competency.

Study 6 explored immigrant-serving agencies’ roles as partner organizations to dementia service institutions and in facilitating access to dementia diagnosis and care services and supports provided by dementia service institutions. Findings from focus groups with older immigrant adults showed that the immigrant-serving agency connected with immigrant clients and was able to engender trust and provide culturally responsive health information as well as support in navigating the health system. The immigrant-serving agency lacked specific knowledge on dementia (a barrier to aligning their messages with clients’ perceptions).

#### 3.3.3. Community Level

Study 4 addressed community-level relations by uncovering the role of settlement service organizations as broker organizations supporting a network of community-based services that meet the primary healthcare needs of immigrants. For example, settlement services in this study function as brokers by acting as a hub for health information for immigrant clients, by being a source of referral to primary care services for immigrant families, and by fostering collaboration in service delivery to high-needs immigrant families while building system competencies with partners. Further, compared to other service-sector organizations, immigrant settlement services in this study were found to have the greatest numbers of strong ties to partners in their community network. Barriers for settlement service organizations to assume the broker role included funding issues or capacity-building resource issues.

#### 3.3.4. Multiple Levels

While no study examined relations solely at the society level, a total of five studies addressed healthcare issues at multiple levels. Study 1 covers individual, relationship and community levels. Specifically, it examined the relationship between recent immigrants’ self-perceived mental health and social supports available for them. Findings revealed that each settlement service organization provided social support by engaging in private meetings with clients or providing referrals to community agencies, local organizations for psychological/clinical counseling, or community group programs. Settlement service organizations in small urban centers offered more tangible social supports compared to those in large urban settings; these included resources to primary healthcare services such as appointment accompaniment and additional referrals to healthcare service providers outside the clients’ community. A limiting factor to being able to provide these social supports was the amount of dedicated staff time needed.

Study 10 explored the successes of community health workers at the individual, relationship and community levels in facilitating access to healthcare for recent immigrants and refugees through a case study of a Multicultural Health Broker Co-op collaborating with a health services public health unit. Findings from this study show the complementary role that multicultural health brokers and community health workers fill within the health system. Multicultural health brokers and community health workers work towards breaking down barriers (such as language, economic conditions, systematic discrimination) to accessing healthcare services for immigrant and refugee families. For example, multicultural health brokers/community health workers accompany clients to appointments or clinics, organize community development initiatives, and offer educational outreach programs on chronic disease prevention and management. A challenge for these community health workers and multicultural health brokers is not being formally recognized as part of the human health resource workforce.

As another study considering the individual, relationship, and community levels, study 7 assessed the impact of a refugee health clinic’s partnership with a local refugee receiving center and community providers on referrals and wait times. The refugee health clinic model uses integrating mechanisms to deliver culturally appropriate and responsive primary care. Within this partnership model, gateway services are provided by the local receiving center’s case workers/settlement workers and professionals from the family practice (e.g., nurse, resident physician). The health clinic delivers comprehensive care via family physicians; interpreters (if needed) are funded by the refugee receiving center. The model also includes ancillary services that are delivered in a community setting by providers willing to treat government-assisted refugees. Study findings demonstrated a 30% decrease in wait times for an appointment with a healthcare provider; an 18% increase in government-assisted refugees securing a permanent family doctor within a year after arrival; and almost a doubling of referrals to non-physician primary healthcare providers (e.g., dentists, optometrists). The study notes that this partnership model is built on goodwill; no formal contracts or funding beyond regular settlement services support was pursued.

Study 2 describes the Asylum Seeker Integrated Healthcare Pathway, an intervention influencing factors on the relationship and community levels, created to improve linkage to health services for asylum seekers newly released from detention. The Pathway consists of settlement support agencies in partnership and collaboration with local primary and emergency healthcare services in Melbourne. The Pathway intervention embeds a clinical health screening and triage process, facilitated by settlement support agencies, into existing community orientation programs for asylum seekers. Findings showed agencies supporting the coordination of healthcare appointments; assisting clients to appointments; and linking clients with culturally responsive care options. Through this initiative, clients had timely access to services. The study noted that an ongoing consideration for the success of this intervention is the capacity of the primary healthcare practices to meet the unique health needs of asylum seekers.

Study 9 addressed individual, relationship, community, and society levels. It discussed the community health worker role in a Multicultural Health Broker Co-op (MCHB Co-op) that supports at-risk immigrant refugee women and their families by contributing to their settlement and integration into communities. The study aimed to better understand the health promotion functions and programs of the MCHB Co-op model and health brokers practice and found that both are able to offer a variety of supports to immigrant and refugee families; the Co-op provides educational support to help them realize their rights in order to overcome access to care barriers as patients (e.g., asking doctors for health information), as program users (e.g., seeking services from the health system), and as citizens (e.g., voicing their concerns to policy decision-makers). Two factors that could have negative implications on the community capacity-building programs and the services delivered by the MCHB Co-op were unstable funding and heavy caseloads.

## 4. Discussion

Findings from this scoping review show that local non-medical immigrant settlement organizations in the 10 articles had established approaches/interventions to support immigrants’ access to primary healthcare services. Further, most of the studies show that mental health support was an important component of the established approaches/interventions. These include: connecting to healthcare services and/or collaborating with health sector institutions; providing health promotion programs; undertaking community capacity-building and policy advocacy activities; and providing ‘on the ground’ assistance to clients. Using the social-ecological model to map these approaches [18], we found that most occurred at multiple levels (individual, relationship, community, and/or society) (study 1, 2, 7, 9, 10); two studies applied approaches/interventions that influence factors to access healthcare services at the individual level (study 3, 8); two studies applied approaches/interventions that influence factors to access healthcare services at the relationship level (study 5, 6); one study applied approaches/interventions that influence factors to access healthcare services at the community level (study 4); and no studies applied approaches/interventions at solely the society level (this may be because societal factors that favor or impair healthcare access, such as health/economic/social policies, require significant intersectoral action to reduce socioeconomic inequalities to healthcare service access).

Out of the 10 studies included in this review, eight were Canadian; this highlights the uniqueness of the Canadian settlement model and long experience of the settlement sector in collaborating and partnering with organizations both within the sector and across other sectors. A case in point is the creation and deployment of LIPs since 2008, and the work they have done to coordinate service provision, for example, by launching numerous innovative initiatives, some focused on primary healthcare, during Canada’s Syrian Refugee Resettlement Initiative in 2015−2016 [15].

As seen in this scoping review, local non-medical immigrant settlement organizations support immigrant access to primary healthcare; however, the scope and quality of services available to immigrants may not be uniform across settlement organizations. Settlement service organizations in Canada receive funding from multiple sources including the federal and provincial governments [40], which can influence or limit a settlement organization’s mandate and/or resources. Further, a lack of responsive and forward-planning federal policy coordinating the provision of settlement services can also lead to disparities in the quality and range of settlement service organization programming between regions where settlement organizations operate [41,42]. Although community-based organizations often enjoy functioning with less bureaucratic control and with organizational structures that can be adapted to social/economic/political contexts to allow for more tailored programming to address inequities and specific marginalized population needs within their communities, these organizations often face challenges of overextended staff with limited resources/funding [43]. Despite the challenges, these organizations are uniquely positioned ‘on the ground’, where they are able to identify the healthcare needs of immigrant populations within the community and closely work with clients (e.g., via community health workers) to address health concerns (e.g., education programming internal to the settlement organization), support healthcare system navigation, provide referrals to health services, and partner/collaborate with health sector institutions to delivery health programs and initiatives [44]. These functions and roles are consistent with literature outlining successful organizational ‘building blocks’ to improve access to primary healthcare for marginalized populations [45].

Local non-medical immigrant settlement organizations may not be structurally or financially able to take on extensive activities to increase access to primary healthcare themselves, however, they have a place in the health system. Consistent with previous literature, community-based organizations are increasingly recognized for their importance in primary healthcare; their unique closeness to immigrant populations and their ability to understand and respond to these populations makes them a valuable partner, source of knowledge, and gateway to marginalized populations for primary healthcare providers and institutions [3,46]. Health systems and services could benefit from including these community-based organizations in their future plans to address the health needs of immigrant populations. There is a need for in-depth research on community collaboration for health equity.

### 4.1. Implications for Research

This scoping review contributes to the literature by making visible the work that local non-medical immigrant settlement organizations do to advance health equity. Nevertheless, more international consensus is needed on terms for community settlement programs and more research on the collaborative relationships that exist or do not exist between community programs and community primary healthcare clinics to explore the impact on health outcomes for immigrant populations. Future research and development in this area is needed to better understand the impact of local non-medical immigrant settlement organizations involved in healthcare planning and service delivery on reducing barriers to access in order for primary care services to reach marginalized, high-need immigrant populations. Further studies could also look at what local non-medical immigrant settlement organizations do to advance health equity in areas linked to the determinants of health, which influence the health outcomes of individuals. This work requires multi-sector response, including but not limited to dealing with migration status, food security, and discrimination. Lastly, it would be beneficial for future research to build on this review by specifically considering the addition of gray literature from different countries and their local non-medical immigrant settlement organizations; gray literature can be very current, detailed, geographically specific, and in essence provide a rich and balanced picture of approaches/interventions to complement these foundational review findings.

### 4.2. Strengths and Limitations of This Scoping Review

Strengths of this scoping review include its methodological approach—that is, using a predefined protocol aligned with Arksey and O’Malley’s framework and the JBI guidance, along with the use of predefined eligibility criteria by two reviewers when selecting the articles [19,20]. There also are a number of limitations, however, that ought to be noted: reviewers did not appraise the quality of the evidence; the scoping review was limited to published peer-reviewed studies; the broad concept of local non-medical immigrant settlement organizations may not have captured all organizations that perform local settlement work with immigrants; the language and terms used in the search may not have been internationally used, and thus, we predominantly identified Canadian-only publications; and many of the studies lacked details on organizational structure, capacity, and programming, which would have been useful to better understand how these organizations are able to support access to primary care.

## 5. Conclusions

Using a social-ecological approach, this scoping review mapped and highlighted current approaches/interventions relating to how local non-medical immigrant settlement organizations support access to primary healthcare services for immigrant populations. Although these findings may not be globally representative and, therefore, not generalizable, they suggest that these organizations are able to support access to primary healthcare services by collaborating with health sector partners in the community network, connecting clients to health services and service providers, advocating for immigrant health, providing educational programming, and also taking on community development/mobilization and advocacy activities to promote access to healthcare. Including these local non-medical immigrant settlement organizations in healthcare planning and service delivery may provide more scope to respond to and reach marginalized, high-need immigrant populations. Strategies to encourage the involvement of local non-medical immigrant settlement organizations in healthcare planning, implementation, and service delivery are needed. Although most of the articles in this review were Canadian, other countries may consider adapting the approaches and interventions identified to their context and needs. As a next step, we recommend a critical assessment of each identified approach/intervention to better understand the feasibility to implement the necessary elements (e.g., human resources required, cost, acceptability of approach), and the extent of its effectiveness. A critical assessment can help relevant stakeholders decide if the identified approaches/interventions in this review are worth adapting.

## Figures and Tables

**Table 1:** Selection Criteria for studies included in the review.

Inclusion Criteria	Description	Exclusion Criteria
Population	Asylum seeker (16 years and older) “Someone whose request for sanctuary has yet to be processed” [23].	All populations other than immigrants, refugees and asylum seekers of all ages. Exclude for feasibility reasons the following: undocumented migrants, transient migrant workers, foreign temporary workers, and foreign students.
	Refugee (16 years and older) “Someone who is unable or unwilling to return to their country of origin owing to a well-founded fear of being persecuted for reasons of race, religion, nationality, membership of a particular social group, or political opinion” [24].	
	Immigrant (16 years and older) “Immigrant refers to a person who is, or who has ever been, a landed immigrant or permanent resident. Such a person has been granted the right to live in Canada permanently by immigration authorities. Immigrants who have obtained Canadian citizenship by naturalization are included in this group.” [25].	
Intervention/Phenomena of Interest	Non-medical (nonclinical) local immigrant settlement organizations that support immigrant population’s access to healthcare services (i.e., healthcare being primary health care or clinical care services)	All other organizations
Context	Industrialized countries with demographics and/or country characteristics comparable to Canada that are ranked on health care system performance by the Commonwealth Fund: Australia, Canada, France, Germany, Netherlands, New Zealand, Norway, Sweden, Switzerland, UK, USA [22,26].	All other countries
Research Type	Research publications (methods, data and analysis) quantitative, qualitative, or mixed-method documents published in peer-reviewed publications	Exclude literature reviews, gray literature
Year of Publication	Last 8 years (since March 2013)	Prior to the last 8 years
Language of Publication	All languages	No exclusion

**Table 2:** Characteristics of included studies.

Study #	Authors/Year	Source Origin	Study Design	Local Non-Medical Settlement Organization	Study Population	Outcome: Approach to Support Access to Primary Healthcare Services for Immigrants	Social-Ecological Model Level
**1**	Chadwick et al., 2015	Canada	quantitative survey analysis; qualitative interviews	settlement service organizations	recent immigrants in large or small urban centers	connects to healthcare services/collaborates with health sector institutions (via resources to services such as appointment accompaniment and referrals to external community service providers, delivery of group programs)	Individual, relationship, community
**2**	Cheng et al., 2019	Australia	community-based intervention development	local settlement support agencies	asylum seekers newly released from detention in South Eastern Melbourne	connects to healthcare services/collaborates with health sector institutions (via the development of the asylum integrated healthcare pathway)	relationship, community
**3**	Frost et al., 2018	United States	exploratory, post hoc, single-group only research design with interviews	local refugee resettlement agency	Burmese-speaking refugee women in Houston Texas	provides health promotion programs (via health education program)	individual
**4**	Isaacs et al., 2013a	Canada	qualitative case study includes survey and interviews	community-based organization	recent immigrant families in an urban center in Atlantic Canada	connects to healthcare services/collaborates with health sector institutions (via role as broker organization)	community
**5**	Issacs et al., 2013b	Canada	qualitative case study includes surveys and interviews	community-based organization	recent immigrants and/or families in an urban community in Atlantic Canada	connects to healthcare services/collaborates with health sector institutions (via cultural competence trust with network)	relationship
**6**	Koehn et al., 2019	Canada	qualitative case study includes focus groups and interviews	immigrant-serving agencies	Punjabi and Korean-speaking older immigrants	connects to healthcare services/collaborates with health sector institutions (via capacity to connect with services and provide culturally responsive health information and navigational support)	relationship
**7**	McMurray et al., 2014	Canada	before/after repeated survey design	local receiving center	government-assisted refugees (primarily coming from Northwest Africa, the Middle East, and Southeast Asia) in Ontario	connects to healthcare services/collaborates with health sector institutions (via partnership between a dedicated health clinic, a local reception center, and community providers)	individual, relationship, community
**8**	Salami et al., 2019	Canada	qualitative descriptive design includes interviews, focus groups	immigrant-serving agencies	immigrants, refugees in Alberta	connects to healthcare services/collaborates with health sector institutions (by identifying client needs, referring clients to specialized mental health services)	individual
**9**	Torres et al., 2013	Canada	qualitative and quantitative case study includes direct observation, interviews, document and database analysis	community-based organization	at-risk immigrant and refugee women and their families in Edmonton	provides health promotion programs (e.g., perinatal program intervention through innovative Multicultural Health Brokers Co-op); undertakes community capacity building and policy advocacy activities (e.g., perinatal program intervention through innovative Multicultural Health Brokers Co-op)	individual, relationship, community, society
**10**	Torres et al., 2014	Canada	qualitative and quantitative case study includes direct observation, interviews, document and database analysis	community-based organization	new immigrants, refugees, and their families in Edmonton	connects to healthcare services/collaborates with health sector institutions (via role as cultural health broker through innovative Multicultural Health Brokers Co-op); provides health promotion programs (via educational outreach on disease management through innovative Multicultural Health Brokers Co-op); provides ‘on the ground’ assistance to clients (e.g., transport to clinics, accompanies clients to doctors appointments when language difficulties are present)	individual, relationship, community

Note: Studies 4 and 5 derive from the same research and research team but have different objectives. Studies 9 and 10 derive from the same research but also have different objectives.

## Data Availability

The Supplementary Excel File (S3) holds information extracted from studies used in this review.

## Supplementary Materials

The following supporting information can be downloaded at: https://www.mdpi.com/article/10.3390/ijerph19063616/s1. The following are available upon request from the corresponding author: File S1: Preferred Reporting Items for Systematic reviews and Meta-Analyses extension for Scoping Reviews (PRISMA-ScR) Checklist; File S2: Search Strategies for all Databases; File S3: Data Extraction Sheet.
