# Supplementary material for: How Are Non-Medical Settlement Service Organizations Supporting Access to Healthcare and Mental Health Services for Immigrants: A Scoping Review
Source: Int J Environ Res Public Health. 2022 Mar 18;19(6):3616. doi: 10.3390/ijerph19063616 (PMC8956042; doi:10.3390/ijerph19063616)
Supplement: Supplementary file 1 [file ijerph-19-03616-s001.zip › ijerph-1601108-supplementary materials- file S2.pdf]

File S2. Search Strategies for all Databases

Search Strategy: PsycInfo

1. immigrant*.ti,ab.
2. Immigration/
3. immigrat*.ti,ab.
4. refugee*.ti,ab.
5. Human Migration/ or Refugees/
6. (foreign adj1 born).ti,ab.
7. (foreign adj1 national*).ti,ab.
8. (asylum adj1 seeker*).ti,ab.
9. Asylum Seeking/
10. migrant*.ti,ab.
11. 1 or 2 or 3 or 4 or 5 or 6 or 7 or 8 or 9 or 10
12. coalition*.ti,ab.
13. Community Services/ or Collaboration/
14. partnerships.ti,ab.
15. partnership.ti,ab.
16. Cooperation/
17. network.ti,ab.
18. networks.ti,ab.
19. groups.ti,ab.
20. group.ti,ab.
21. organization.ti,ab.
22. organizations.ti,ab.
23. organisations.ti,ab.
24. organisation.ti,ab.
25. Nonprofit Organizations/ or Religious Organizations/ or Faith Based Organizations/
26. service.ti,ab.

27. services.ti,ab.

28. collaborat*.ti,ab.

29. Teams/

30. institute.ti,ab.

31. institutes.ti,ab.

32. institutions.ti,ab.

33. institution.ti,ab.

34. center.ti,ab.

35. centers.ti,ab.

36. centre.ti,ab.

37. centres.ti,ab.

38. 12 or 13 or 14 or 15 or 16 or 17 or 18 or 19 or 20 or 21 or 22 or 23 or 24 or 25 or 26 or 27 or 28 or 29 or 30 or 31 or 32 or 33 or 34 or 35 or 36 or 37

39. local*.ti,ab.

40. Community Involvement/

41. communit*.ti,ab.

42. Communities/

43. civic.ti,ab.

44. region*.ti,ab.

45. city.ti,ab.

46. cities.ti,ab.

47. Urban Environments/

48. municipal*.ti,ab.

49. integrated services/ or outreach programs/

50. 39 or 40 or 41 or 42 or 43 or 44 or 45 or 46 or 47 or 48 or 49

51. health*.ti,ab.

52. Health Promotion/ or Preventive Health Services/ or Public Health/ or Community Health/ or Health Care Access/ or Health/ or Public Health Services/

53. equit*.ti,ab.

54. Equity/

55. inequit*.ti,ab.

56. Health Care Policy/ or Health Care Services/ or Health Disparities/

57. (health adj1 service*).ti,ab.

58. Community Mental Health/ or Mental Health Services/ or Health Care Delivery/ or Health Care Utilization/ or Community Mental Health Services/

59. (primary adj1 care).ti,ab.

60. (primary adj1 healthcare).ti,ab.

61. (primary adj1 health adj1 care).ti,ab.

62. Primary Health Care/

63. medicine.ti,ab.

64. medical.ti,ab.

65. (clinical adj1 care).ti,ab.

66. 51 or 52 or 53 or 54 or 55 or 56 or 57 or 58 or 59 or 60 or 61 or 62 or 63 or 64 or 65

67. canada.ti,ab.

68. (united adj1 kingdom).ti,ab.

69. france.ti,ab.

70. germany.ti,ab.

71. (united adj1 states).ti,ab.

72. switzerland.ti,ab.

73. sweden.ti,ab.

74. australia.ti,ab.

75. (new adj1 zealand).ti,ab.

76. netherlands.ti,ab.

77. norway.ti,ab.

78. 67 or 68 or 69 or 70 or 71 or 72 or 73 or 74 or 75 or 76 or 77

79. 11 and 38 and 50 and 66 and 78

80. limit 79 to last 8 years

Search Strategy: CINAHL

#	Query	Limiters/Expanders
S1	"(immigrant*) OR (immigrat*) OR (refugee*) OR (foreign born) OR (foreign national*) OR (migrant*) OR (asylum seeker*)" OR (MH "Transients and Migrants") OR (MH "Immigrants") OR (MH "Emigration and Immigration")	Expanders<br>- Apply related words; Apply equivalent subjects<br>Search modes<br>-Boolean/Phrase
S2	"(local*) OR (communit*) OR (civic) OR (region*) OR (city) OR (cities) OR (municipal*)" OR (MH "Regional Centers") OR (MH "Community Health Services")	Expanders<br>- Apply related words; Apply equivalent subjects<br>Search modes<br>-Boolean/Phrase
S3	(MH "Great Britain") OR(Canada) OR (MH "Canada") OR (United States) OR (MH "United States") OR (France) OR(MH "France") OR(Germany) OR (MH "Germany") OR (United Kingdom) OR(Switzerland) OR (MH "Switzerland") OR(Sweden) OR (MH "Sweden") OR (Australia)OR (MH "Australia") OR(New Zealand) OR (MH "New Zealand") OR(Netherlands) OR (MH "Netherlands") OR(Norway) OR (MH "Norway") OR (MH "United Kingdom") OR(MH "Virgin Islands of the United States") OR (MH "Southwestern United States") OR (MH "Southeastern United States") OR (MH "Northwestern United States") OR (MH "Midwestern United States")	Expanders<br>- Apply related words; Apply equivalent subjects<br>Search modes<br>-Boolean/Phrase
S4	(MH "Community Health Centers") OR (coalition*) OR (partnership*) OR (network*) OR (group*) OR (organization*) OR (service*) OR (collaborat*) OR (institut*) OR (center*) OR	Expanders<br>- Apply related words; Apply equivalent subjects<br>Search modes<br>-Boolean/Phrase

	(centre*)" OR (MH "Health Maintenance Organizations") OR (MH "Rural Health Centers")	
S5	(MH "Primary HealthCare") OR (MH "Alternative Health Facilities") OR (MH" Health Care Delivery, Integrated") OR (health*) OR (equit*) OR (inequit*) OR (health service*) OR (primary care) OR (primary healthcare) OR (primary health care) OR (health care) OR (medicine) OR (medical) OR (clinical care) OR (MH "Multidisciplinary Care Team") OR (MH "Rural Health Personnel") OR (MH "Healthcare Disparities") OR (MH "Health Care Delivery")OR (MH "Medical Care")OR (MH "Tertiary Health Care") OR (MH "Secondary Health Care") OR (MH "Preventive Health Care") OR (MH "Outcomes (HealthCare)") OR (MH "Health Services Accessibility") OR (MH "Community Mental Health Services") OR (MH "National Health Programs") OR (MH "Physicians, Family") OR (MH "Community Health Centers") OR (MH "Adolescent Health Services") OR (MH "Ancillary Services, Hospital") OR (MH "Rural Health Services") OR (MH "Child Health Services") OR (MH "Emergency Medical Services") OR (MH "Community Health Services") OR (MH "School Mental Health Services") OR (MH "Urban Health Services") OR (MH "Mental Health Services") OR (MH "Health Services") OR (MH "Allied Health Organizations") OR (MH "Multiskilled Health Practitioners") OR (MH "Cancer Care Facilities") OR (MH	Expanders<br>- Apply related words; Apply equivalent subjects<br>Search modes<br>-Boolean/Phrase

	"Hospitals, Psychiatric") OR (MH "Mental Health Personnel") OR (MH "Hospitals, Public") OR (MH "Hospitals, Pediatric") OR (MH "Specialties, Allied Health") OR (MH "Psychiatric Care") OR (MH "Health Promotion") OR (MH "Health Policy") OR (MH "Community Health Workers")	
S6	S1 AND S2 AND S3 AND S4 AND S5	Limiters<br>- Published Date:20130501-20210531<br>Expanders<br>- Apply related words; Apply equivalent subjects<br>Search modes<br>-Boolean/Phrase

Search Strategy: MEDLINE

1. immigrant*.ti,ab.

2. "Emigrants and Immigrants"/

3. immigrat*.ti,ab.

4. "Emigration and Immigration"/

5. refugee*.ti,ab.

6. Refugees/

7. (foreign adj1 born).ti,ab.

8. (foreign adj1 national*).ti,ab.

9. "Transients and Migrants"/

10. (asylum adj1 seeker*).ti,ab.

11. migrant*.ti,ab.

12. 1 or 2 or 3 or 4 or 5 or 6 or 7 or 8 or 9 or 10 or 11

13. coalition*.ti,ab.

14. Health Care Coalitions/

15. partnerships.ti,ab.

16. partnership.ti,ab.

17. network.ti,ab.

18. networks.ti,ab.

19. group.ti,ab.

20. groups.ti,ab.

21. organization.ti,ab.

22. organizations.ti,ab.

23. organisations.ti,ab.

24. organisation.ti,ab.

25. service.ti,ab.

26. services.ti,ab.

27. collaborat*.ti,ab.

28. institute.ti,ab.

29. institutes.ti,ab.

30. institution.ti,ab.

31. institutions.ti,ab.

32. center.ti,ab.

33. centers.ti,ab.

34. centre.ti,ab.

35. centres.ti,ab.

36. 13 or 14 or 15 or 16 or 17 or 18 or 19 or 20 or 21 or 22 or 23 or 24 or 25 or 26 or 27 or 28 or 29 or 30 or 31 or 32 or 33 or 34 or 35

37. local*.ti,ab.

38. communit*.ti,ab.

39. Community Health Services/

40. civic.ti,ab.

41. region*.ti,ab.

42. city.ti,ab.

43. cities.ti,ab.

44. municipal*.ti,ab.

45. 37 or 38 or 39 or 40 or 41 or 42 or 43 or 44

46. health*.ti,ab.

47. Health Care Sector/ or Mental Health Services/ or Health Services/

48. Health Policy/

49. equit*.ti,ab.

50. inequit*.ti,ab.

51. Health Status Disparities/ or Health Equity/ or Health Services Accessibility/ or "Delivery of Health Care"/

52. Public Health/

53. Health Promotion/

54. (health adj1 service*).ti,ab.

55. (primary adj1 care).ti,ab.

56. (primary adj1 healthcare).ti,ab.

57. (primary adj1 health adj1 care).ti,ab.

58. medicine.ti,ab.

59. medical.ti,ab.

60. (clinical adj1 care).ti,ab.

61. 46 or 47 or 48 or 49 or 50 or 51 or 52 or 53 or 54 or 55 or 56 or 57 or 58 or 59 or 60

62. Canada.ti,ab.

63. (united adj1 states).ti,ab.

64. france.ti,ab.

65. germany.ti,ab.

66. (united adj1 kingdom).ti,ab.

67. switzerland.ti,ab.

68. sweden.ti,ab.

69. australia.ti,ab.

70. (new adj1 zealand).ti,ab.

71. netherlands.ti,ab.

72. norway.ti,ab.

73. Germany, East/ or Germany/ or Germany, West/

74. South Australia/ or Australia/ or Western Australia/

75. Caribbean Netherlands/ or Netherlands/ or Netherlands Antilles/

76. 62 or 63 or 64 or 65 or 66 or 67 or 68 or 69 or 70 or 71 or 72 or 73 or 74 or 75

77. 12 and 36 and 45 and 61 and 76

78. limit 77 to last 8 years

Search Strategy: Social Services Abstracts

Name:
Social Services Abstract May 31 2021Edit name
Searched for:
(AB,TI(immigrant* OR emigrant* OR immigrat* OR refugee* OR "foreign born" OR ("foreign national" OR "foreign nationalities" OR "foreign nationality" OR "foreign nationals") OR transient* OR migrant* OR ("asylum seeker" OR "asylum seekers")) OR MAINSUBJECT.EXACT("Immigration") OR MAINSUBJECT.EXACT("Immigrants") OR MAINSUBJECT.EXACT("Migrants") OR MAINSUBJECT.EXACT("Refugees")) AND (AB,TI(coalition* OR partnership* OR network* OR group* OR organization* OR organisation* OR service* OR collaborat* OR institut* OR center* OR centre* OR local* OR communit* OR civic OR region* OR city OR cities OR municipal*) OR MAINSUBJECT.EXACT("Nonprofit Organizations") OR MAINSUBJECT.EXACT("Community Organizations") OR MAINSUBJECT.EXACT("Organizations (Social)") OR MAINSUBJECT.EXACT("Nongovernmental Organizations")) AND (AB,TI(health* OR equit* OR inequit* OR "accessibilit*" OR "primary care" OR medicine OR medical OR "clinical care") OR MAINSUBJECT.EXACT("Health Care Utilization") OR MAINSUBJECT.EXACT("Mental Health Services") OR MAINSUBJECT.EXACT("Health Care Services") OR MAINSUBJECT.EXACT("Health Policy") OR MAINSUBJECT.EXACT("Primary Health Care") OR MAINSUBJECT.EXACT("Health") OR MAINSUBJECT.EXACT("Health Care Services Policy") OR MAINSUBJECT.EXACT("Community Mental Health") OR MAINSUBJECT.EXACT("Public Health")) AND AB,TI(Canada OR "United States" OR France OR Germany OR "United Kingdom" OR "Britain" OR Switzerland OR Sweden OR Australia OR Norway OR "New Zealand" OR Netherlands) AND pd(20130501-20210531)

Databases:

- Social Services Abstracts
